# Warming and Change in Ocean Productivity Alter Phenology of an Expanding Loggerhead Population in Cabo Verde

**DOI:** 10.3390/ani16040552

**Published:** 2026-02-11

**Authors:** Fitra Arya Dwi Nugraha, Kirsten Fairweather, Artur Lopes, Anice Lopes, Berta Renom, Rebekka Allgayer, Albert Taxonera, Christophe Eizaguirre

**Affiliations:** 1 Department of Biology, School of Biological and Behavioural Sciences, Queen Mary University of London, London E1 4NS, UK; f.a.d.nugraha@qmul.ac.uk; 2 Associação Projeto Biodiversidade, Santa Maria 4111, Sal, Cabo Verde; kirsten.fairweather@projectbiodiversity.org (K.F.); artur.lopes@projectbiodiversity.org (A.L.); anice.lopes@projectbiodiversity.org (A.L.); berta.renom@projectbiodiversity.org (B.R.); albert.taxo@projectbiodiversity.org (A.T.); 3 Associação de Defesa de Tartarugas Marinhas e seu Ambiente—SOS Tartarugas, Santa Maria 4111, Sal, Cabo Verde; 4 School of Biological Sciences, University of Aberdeen, Aberdeen AB24 2TZ, UK; rebekka.allgayer@rspb.org.uk; 5 Royal Society for the Protection of Birds Centre for Conservation Science, The Lodge, Sandy SG19 2DL, UK

**Keywords:** climate change, phenology, sea turtles, ectotherm, capital breeding, reproduction, migration

## Abstract

Climate warming can alter reproductive timing of species, yet the capacity for phenological adjustment in long-lived species, particularly marine ones, remains elusive. Using 17 years of monitoring data from one of the largest loggerhead turtle (*Caretta caretta*) populations, we investigated the environmental drivers of reproductive phenology and output. We found that warmer sea surface temperatures (SST) in both the feeding ground and the nesting ground advanced the start, peak, and end of the nesting season. We provide evidence for waves of arrival at the nesting ground, suggesting more turtles produce fewer clutches than previously thought. Inter-nesting intervals were shorter during episodes of higher SST, particularly in larger females, likely underpinned by metabolic scaling variation in reproductive pacing. Conversely, remigration intervals lengthened over time in all size classes, reflecting the detected continuous decrease in productivity in the feeding ground. As a result of reduced ocean productivity, both clutch size and clutch frequency also declined over the study period. Moreover, the declining trend in body size further reduces reproductive output, as smaller females produce smaller clutch sizes. Overall, we show that sea turtle population dynamics correlate with environmental parameters. The sustained decline in reproductive output underscores the need to mitigate the impacts of climate warming on the foraging area to safeguard this population, which, given its size, holds global significance.

## Simple Summary

The diverse responses of sea turtles to climate warming highlight the need for population-specific studies. Here, we used long-term monitoring data from one of the largest loggerhead turtle nesting populations in the world to investigate the reproductive phenology and output in relation to climate warming. We found that warmer years were associated with earlier phenology and longer nesting seasons. At the same time, increased temperatures reduced ocean productivity, which prolonged foraging periods and consequently decreased clutch frequency and size. This decline in reproductive output may ultimately compromise population resilience and slow recovery in the face of ongoing climate warming.

## 1. Introduction

Climate change reshapes biological systems, influencing the behaviour, physiology, and distribution of many species across the globe [1]. Organisms may respond to these changes through various adaptive mechanisms, including shifts in the timing and the location of life-history events, or change in genetic composition [2,3]. Among these, phenological adjustments, defined as the timing of biological activities within and across years, are among the most pervasive [4,5]. Such changes occur across diverse taxa and life-history events, such as the timing of plant flowering and insect flight [6], bird breeding [7], amphibian breeding [8], fish-spawning [9], and mammals parturition [10].

Failure to track environmental change can lead to phenological mismatch, where critical life-history events no longer align with peak resource availability, with consequences for reproductive success and population persistence [11,12,13]. In sea turtles, however, phenological mismatch with food resources is rarely the proximate concern [14]. Instead, nesting phenology is tightly coupled to thermal conditions, as local climatic temperatures influence the timing of nesting, incubation duration, hatchling success, and sex ratios [14,15]. Sea turtles show evidence of phenological plasticity, though the extent of these shifts varies geographically [16,17]. Changes in nesting timing are often associated with sea surface temperatures (SST), yet both the direction and strength of the relationship, and whether it reflects SST from breeding or foraging grounds, or particular phenological metric (e.g., start, median, or end of nesting) differ among species and populations [18,19,20,21]. This context dependence highlights the need for population-specific studies to understand the environmental drivers of nesting phenology and improve local predictions under future climate scenarios.

To initiate nesting, sea turtles undertake migrations from foraging grounds that can be located thousands of kilometres away from breeding sites [22]. These migrations are thought to be triggered by temperature cues [23], suggesting that temperature variations can modulate both migration and nesting activity. However, conditions at breeding grounds also play a role. Some studies suggest that turtles arrive early to mate and complete egg development, with nesting subsequently timed to coincide with favourable environmental conditions [24,25,26].

Within nesting seasons, the inter-nesting interval varies among species and correlates with SST [27,28,29,30,31]. Although previously assumed to reflect yolk development time, vitellogenesis is completed prior to arrival at nesting beaches [32,33,34], and females can store sperm throughout the season [35,36]. Inter-nesting intervals therefore likely reflect the final stages of egg formation [37] and metabolic recovery [38]. Thermal inertia in larger individuals may buffer against temperature fluctuations, whereas smaller turtles may respond more rapidly to temperature change [39,40]. Thus, both body size and environmental factors interact to shape the timing of clutch production.

Beyond timing, reproductive output, including clutch size, is influenced by the availability of resources at foraging sites [41]. As capital breeders, sea turtles rely on energy accumulated prior to migration; access to rich foraging habitats enhances energy storage, facilitates migration and supports greater reproductive investment [42]. Productive foraging areas may also shorten the time needed to reach energetic thresholds, thereby influencing remigration intervals. Clutch size correlates with body size [43], which in turn reflects foraging success [44,45] and energy budgets [46]. Because resource abundance is itself modulated by temperature [47,48], interactions among temperature, resource acquisition, and body condition may jointly determine reproductive traits such as clutch size, frequency and remigration intervals.

The Cabo Verde archipelago hosts one of the world’s largest loggerhead turtle (*Caretta caretta*) populations, with hundreds of thousands of nests laid annually [49]. Research there has focused on population dynamics [50,51], population genetic structure [52,53], disease ecology [54], habitat vulnerability [55], and feeding ecology [42]. Collectively, these studies highlight the ecological and evolutionary significance of the Cabo Verde rookery and provide essential foundations for conservation. Despite these advances, the influence of climate warming on nesting phenology and reproduction dynamics in this globally important population is poorly understood. To address this gap, we combined long-term monitoring and tagging data to evaluate environmental and biological drivers of nesting timing reproductive output. Specifically, we hypothesize (1) that temperature is associated with phenology; (2) that temperature interacts with female body size to influence inter-nesting intervals; (3) and that foraging productivity interacts with temperature and body size to influence remigration intervals and clutch size.

## 2. Materials and Methods

### 2.1. Study System and Sea Turtle Monitoring

**Figure 1:**
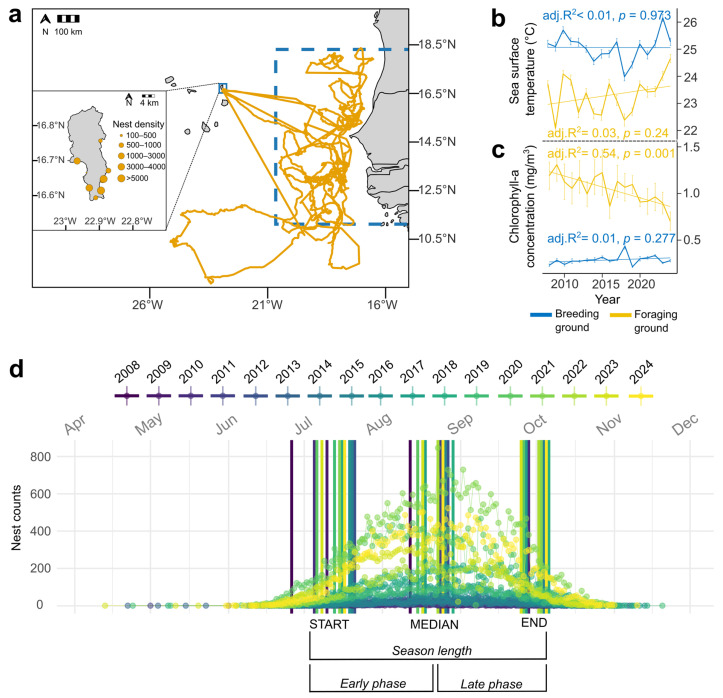
Study location, environmental trends at foraging and breeding grounds, and temporal distribution of nesting. (**a**) Map showing *C. caretta* nesting and foraging areas associated with the Sal Island’s aggregation in Cabo Verde. Orange lines represent satellite-tracked females migrating from Sal Island to their foraging grounds along the West African coast. The blue dashed box indicates the main foraging area from which environmental data were extracted. The inset shows Sal Island, where the breeding beaches are located; environmental data for the breeding ground was derived from this area. Circles denote relative nest density across beaches. (**b**) Interannual variation in seasonal sea surface temperature (SST) at the breeding (blue) and foraging (yellow) grounds. No significant trend was detected for either region. (**c**) Interannual variation in seasonal chlorophyll-a (CHL) concentration, showing a significant decline in the foraging grounds. Seasonal values for the foraging grounds correspond to November of year n to May of year *n* + 1; for breeding grounds, June–October of the same year. (**d**) Daily nest counts and phenology of loggerhead nesting on Sal Island from 2008 to 2024. Each point represents the number of nests recorded per day, with colours distinguishing seasons. Vertical lines mark the 5th, 50th, and 95th percentiles of cumulative nest counts, defining the start, median, and end of each nesting season, respectively. Season duration corresponds to the period between the 5th and 95th percentiles, with the early and the late phases representing the increasing and decreasing portions of the nesting curve.

This study was conducted on Sal Island, Republic of Cabo Verde, located ~600 km off the African coast (Figure 1a). Nightly foot patrols and morning surveys were carried out during nesting seasons, from June to November, between 2008 and 2024. During night patrols, all nests were recorded, regardless of whether a turtle was directly observed. When a nesting turtle was encountered, she was checked for the presence of metal tags or a passive integrated transponder (PIT), and the GPS coordinates of the nesting site was recorded. Untagged turtles were fitted with metal tags on both flippers and/or a new tag (PIT) on the right flipper for the years 2008–2018, and from 2019 on, a new tag (PIT) only. Curved carapace length (CCL) was measured with a tape to the nearest 0.1 cm. From 2008 to 2015, CCL was recorded from notch-to-tip, whereas from 2017 onward, it was measured from notch-to-notch. In 2016, both methods were used, enabling us to calculate the mean difference between measurement methods. To standardize measurements over the full dataset, this mean difference was subtracted from all notch-to-tip values recorded before 2016. Clutch size was recorded each time nests required relocation, either in situ or into a hatchery.

Over 17 years of monitoring, we recorded 178,566 nests and tagged 14,162 individuals. Tagging data were used to derive life-history traits for each individual in each year: (1) neophyte vs. remigrant status, (2) number of clutches per season (clutch frequency), (3) interval between successive clutches (inter-nesting interval), and (4) interval between nesting seasons for remigrants (remigration interval).

### 2.2. Determination of Nesting Phenology and Season Duration

Defining the start and end of the season based on the first and last observed nest might be obscured by the arrival of sporadic nesting females or by the fact that we may miss some activities early and late in the season. To minimize this possible bias, we used a percentile-based approach: the start of the season was defined as the day by which 5% of total nests were laid, the median as the 50th percentile, and the end as the 95th percentile (Figure 1d). Season length was calculated as the number of days between the 5th and 95th percentiles. The start duration (early phase) corresponded to the period between the 5th and 50th percentiles, and the end duration (late phase) between the 50th and 95th percentiles.

### 2.3. Determination of Inter-Nesting and Remigration Interval and Clutch Frequency and Size

The inter-nesting interval was defined as the number of days between successive clutches. We first included all inter-nesting intervals ranging from 9 to 84 days in the analyses. To reduce possible errors from potential misidentification or missed nesting observations, we also performed the same analysis excluding inter-nesting intervals shorter than 9 days and longer than 20 days [27,56]. The remigration interval, on the other hand, refers to the time between two reproductive migrations. Clutch frequency was the total number of clutches laid by an individual within a single nesting season, and clutch size was the mean number of eggs per clutch for that individual. When multiple CCL measurements were available for a turtle in a season, their average was used in the analyses.

### 2.4. Estimated Chronology of Nesting Activities

It is important to identify the chronology of reproduction to identify the right environmental drivers responsible for the specific reproductive traits. Ovulation for the season is induced by mating [57], but whether females mate immediately upon arrival at the nesting grounds in Cabo Verde remain unclear. Ovulation and gravidity likely occur 11–14 days [27,57] before laying eggs. Given that nesting typically begins in June–July (Supplementary Figure S1), females must reach the breeding grounds by mid-May or mid-June to allow sufficient time for ovulation and egg development.

Based on telemetry data from 5 tracked females returning from nesting to foraging grounds (2011 and 2022), migration may last up to two months, implying departure from foraging areas between late March and April (Supplementary Figure S1). Therefore, environmental cues during March–April may influence migration timing.

From this estimated chronology, we formulated the following hypotheses:

1. Nesting phenology: The start of nesting correlates with SST at the foraging grounds during March–April and/or at the breeding ground during May–June. Median nesting dates may correlate with SST at the breeding ground during June–August, and season end with SST at the breeding ground during September–October.
2. Inter-nesting interval: Inter-nesting intervals correlate with SST and chlorophyll-a (CHL) at the breeding grounds and with body size, as egg maturation between clutches is temperature-dependent and potentially size-mediated.
3. Remigration intervals: Remigration intervals correlate with SST and CHL at the foraging grounds (November–May) and body size, as energy accumulation for reproduction depends on resource availability and temperature-driven habitat use. CHL reflects ocean primary productivity and is linked to loggerhead foraging resources, given that higher CHL often indicates greater abundance of planktonic food webs that support their prey [58,59].
4. Clutch Frequency: Clutch frequency correlates with SST at breeding grounds (June–October), CHL at foraging grounds (November–May), and body size, since both temperature and energy reserves can influence the number of clutches per season.
5. Clutch size: Clutch size correlates with SST at breeding grounds, CHL at foraging grounds, body size, and clutch frequency.

### 2.5. Environmental Data

Sea surface temperature (SST) and chlorophyll-a concentration (CHL) were taken from both foraging (11.12–18.31° N, −14.50–−20.66° W) and breeding grounds (16.5–16.9° N, −23.1–−22.7° W). All environmental data were retrieved from Copernicus Marine Service (https://marine.copernicus.eu/). For the phenological analyses, SST data were obtained from https://data.marine.copernicus.eu/product/SST_GLO_SST_L4_NRT_OBSERVATIONS_010_001/description (accessed on 29 January 2025) at a 5 km spatial resolution and CHL data were obtained from https://data.marine.copernicus.eu/product/OCEANCOLOUR_GLO_BGC_L4_MY_009_108/description (accessed on 17 March 2025) at a 4 km spatial resolution. For the inter-nesting interval analyses, daily CHL data were retrieved from https://data.marine.copernicus.eu/product/OCEANCOLOUR_GLO_BGC_L3_MY_009_107/description (accessed on 12 June 2025) at a 4 km spatial resolution and daily SST were obtained from the same sources as SST for phenological analyses.

For remigrant turtles, because we know how long they have stayed in the foraging ground, the mean CHL and SST were calculated for that specific period. For example, if a turtle returned to nest in 2010 after being recorded nesting in 2008, the average CHL and SST during the foraging period were calculated from 1 November 2008 to 31 May 2010. For first-time nesters, however, because the duration of their time in the foraging ground is unknown, we calculate the mean CHL and SST over the four years preceding the year in which they were first recorded nesting. In the inter-nesting intervals analyses, mean CHL and SST were calculated over the interval between the date of the initial observed nesting event and the date of the subsequent observed nesting event.

### 2.6. Statistical Analyses

#### 2.6.1. Temporal Trends and Female Number Estimate

We analyzed the following phenological parameters: start, median, end of season, total duration, start duration, end duration, remigration intervals, inter-nesting intervals, clutch frequency, and clutch size. Temporal trends were assessed using linear regression models (LMs). LMs were also used to examine the relationships between phenological parameters and their environmental predictors. Differences between neophytes and remigrants in inter-nesting intervals, clutch frequency, and clutch size were tested with Wilcoxon rank sum tests.

The number of female nesters was estimated using a rolling window approach. Based on the observed inter-nesting intervals, a 14-day rolling window was applied to the daily nest count data, shifting forward every two days throughout the nesting season. Each window was assumed to represent a distinct set of individuals. For comparison, if the average number of clutches per female is three, the number of individuals can alternatively be estimated using a 28-day rolling window, which allows us to estimate the maximum number of turtles nesting at any given time on the basis of 3 nests per turtle. This approach is then shifted forward every two days across the season to maintain comparability with the 14-day window estimates.

#### 2.6.2. Predictors of Reproductive Parameters

Prior to model fitting, data for remigration interval, inter-nesting interval, clutch frequency, and clutch size were evaluated for linearity, normality, multicollinearity, and homoscedasticity. Linearity was assessed using residual-versus-fitted plots and component-plus-residual plots, normality of residuals was formally tested with the Shapiro–Wilk test, multicollinearity was evaluated using Variance Inflation Factors (VIF), and homoscedasticity was tested with the Breusch–Pagan test. Predictors with high multicollinearity (VIF > 10) were standardized (mean = 0, SD = 1) using the scale() function in R version 4.2.2 [60].

Pairwise Pearson correlations were used to test the correlation among predictors. When strong correlations were detected, we removed the shared variation by retaining residuals from a linear model. For example, sea surface temperature (SST) and chlorophyll-a (CHL) concentration in the feeding grounds were highly correlated; in the remigration interval analysis, we included only the portion of CHL not explained by SST, obtained using the resid() function.

#### 2.6.3. Model Selection

We first fitted LMs for each response variable to evaluate assumptions of linearity, normality, and homoscedasticity. When these assumptions were violated, we selected an appropriate model family based on the distribution and nature of the response variable. For example, positive continuous data were modelled using a Gamma distribution, while discrete counts were modelled using Poisson or negative binomial distributions.

For each response variable, we then fitted a set of candidate-generalized linear mixed models (GLMMs), including fixed effects for SST, CHL, and curved carapace length (CCL), as well as relevant interactions. Turtle identity (ID) was included as a random effect to account for repeated measurements and retained only if the estimated variance was greater than zero; otherwise, we fitted a generalized linear model (GLM) without the random effect.

Candidate models were compared using Akaike’s Information Criterion (AIC), and the model with the lowest AIC was selected as the best-supported model. The significance of fixed effects was assessed using likelihood-ratio tests. For GLMs, we used chi-square tests, whereas for GLMMs, type II likelihood-ratio tests were applied to account for the random effect from turtle ID. To control for multiple testing, Bonferroni-adjusted *p*-values were calculated by multiplying the raw *p*-values by the number of terms tested within each model. All analyses were conducted in R version 4.2.2 [60].

## 3. Results

Some nesting activity was detected as early as February; however, these were either false crawls (abandoned digs) or U-turns, where turtles came ashore but returned to the sea without digging. The earliest recorded nests were in April, and the latest were in November (Figure 1d). Throughout our monitoring period, from 2008 to 2024, there were three nests laid in April and nine in May, but the numbers increased to 2158 in June and jumped to 36,304 in July, peaking at 74,081 and 56,210 in August and September, respectively, and declined to 9771 in October and 30 in November (Supplementary Figure S2). The season length ranged from 75 to 93 days and, overall, 14,162 unique turtles were tagged. Regarding environmental conditions, there were no significant temperature trends in either foraging or breeding ground (Figure 1b); however, a decrease in chlorophyll-a concentrations at the foraging site was significant (linear model, LM, adjusted R^2^ = 0.54, F(1,15) = 19.91, *p* < 0.001; see Figure 1c), suggesting that resource availability may not positively influence reproductive parameters in this population in recent years.

### 3.1. Drivers of Nesting Phenology and Season Length

**Figure 2:**
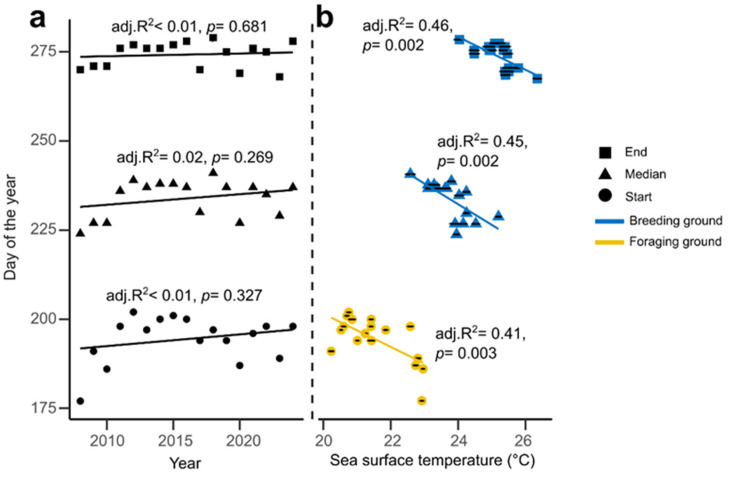
Temporal trends and environmental drivers of nesting phenology. (**a**) Temporal variation in the start (circles), median (triangles), and end (squares) of the nesting season from 2008 to 2024 on Sal Island. No significant long-term trends were detected for any phenological metric. (**b**) Relationship between nesting phenology and seasonal sea surface temperature (SST) in the foraging (yellow) and breeding (blue) grounds. Warmer SSTs were significantly associated with earlier nesting across all phenological metrics, indicating that higher temperatures at either site advance the timing of the season.

We detected no temporal patterns in nesting phenology (Figure 2a). However, the start, median and end dates were each significantly and negatively correlated with SST at breeding or foraging ground (Figure 2b). Specifically, the start of the season correlated with the average SST of March–April in the foraging ground (linear model, LM, adjusted R^2^ = 0.41, F(1,15) = 12.04, *p* = 0.003), the median with SST of June–July in the breeding ground (LM, adjusted R^2^ = 0.45, F(1,15) = 14.15, *p* = 0.002) and the end with SST of June–September in the breeding ground (LM, adjusted R^2^ = 0.46, F(1,15) = 14.58, *p* = 0.002). All these relationships show that warmer temperatures are associated with earlier phenology.

The season length showed no change over the monitored period (LM, adjusted R^2^ = 0.028, F(1,15) = 1.453, *p* = 0.247; see Supplementary Figure S3a) but was best predicted by the seasons’ start (LM, adjusted R^2^ = 0.733, F(1,15) = 44.93, *p* < 0.001; see Supplementary Figure S3b). Specifically, a late start (=when 5% of a season’s nests were counted) was associated with an earlier end (=95% of a season’s nests). The early and late phases showed no significant difference (Wilcoxon signed-rank test, two-sided: V = 43.5, *p* = 0.2121) suggesting that variability in the total of season length is not driven by disproportionate changes in just the early or the late phase of the season. As expected under the evolution of phenology in the face of global warming, warmer years had a faster start (LM, adjusted R^2^ = 0.398, F(1,15) = 11.58, *p* = 0.004; see Supplementary Figure S3c), while higher September SST was associated with extended ends of the nesting seasons (LM, adjusted R^2^ = 0.239, F(1,15) = 6.036, *p* = 0.027; see Supplementary Figure S3d).

### 3.2. Inter-Nesting Intervals

**Figure 3:**
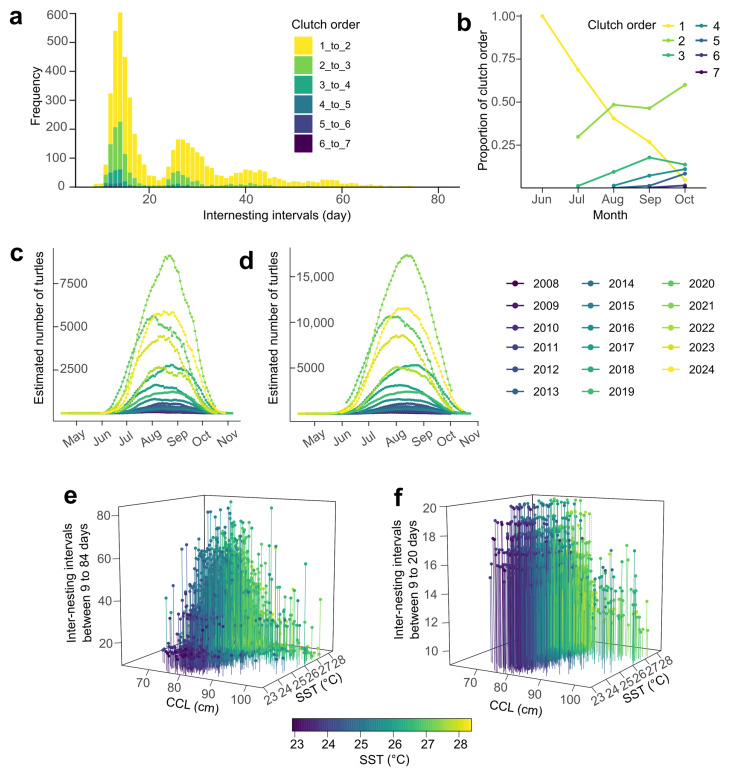
Patterns and predictors of inter-nesting intervals in loggerhead turtle from Sal Island. (**a**) Distribution of inter-nesting intervals (>8 days) within the study period, with bars representing the frequency of intervals grouped by clutch order (1–7). (**b**) Proportion of each clutch order per month, highlighting a decline in first clutches, but with a slower rate of decline between August and September. (**c**,**d**) Estimated number of females using intervals in the 14-day and 28-day windows, respectively. (**e**) Two-way interaction of sea surface temperature (SST) and curved carapace length (CCL) for the whole dataset and (**f**) for intervals between 9 and 20 days.

Based on metal and PIT tags, we found that the inter-nesting intervals ranged from 9 to 84 days, with three clearly identifiable peaks in the distribution, suggestive of highly variable inter-nesting intervals or undetected intermediate nesting events (Figure 3a). Overall, comparing the number of nests to the number of tags detected/deployed per night, across the entire period of the study, patrols tagged on average 22% of daily nesting turtles (Supplementary Figure S4b).

The average inter-nesting intervals was 24.17 ± 13.38 days (mean ± SD, *n* = 5009). The best model linking inter-nesting intervals to environmental parameters included an interaction of SST, CHL, CCL (GLMM, AIC = 9284.4, BIC = 35,633, log-likelihood = −17,806.5, random intercept variance for turtle ID = 0.1649, SD = 0.406; see Supplementary Table S1). The SST at the breeding ground by CCL interaction was significant (Type II Wald χ^2^(1) = 15.26, Bonferroni-adjusted *p* < 0.001; see Figure 3e) with larger turtles showing shorter intervals at higher SST. No other terms were significant.

Restricting the inter-nesting intervals to 9–20 days yielded 14.43 ± 2.01 days (mean ± SD, *n* = 2744). There was no temporal change in the mean inter-nesting interval (LM, adjusted R^2^ = 0.099, F(1,14) = 2.652, *p* = 0.126; see Supplementary Figure S5a), and neophytes exhibited slightly shorter inter-nesting periods than remigrants (Wilcoxon rank sum test, two-sided: W = 290,175, *p* = 0.0021; see Supplementary Figure S5b). Interestingly, the proportion of turtles observed for the first time decreases linearly over the course of the nesting season, until a slower rate is detected between August and September, suggesting a detectable wave of newly arrived turtles (Figure 3b). Using the observed average of inter-nesting intervals, the estimated number of females nesting in Sal was approximately between 9000 and 17,000 at the peak of the nesting activity (Figure 3c,d).

Furthermore, the best model linking inter-nesting intervals to environmental parameters included a three-way interaction between SST, CHL, and CCL (GLMM, AIC = 9284.4, BIC = 9343.4, log-likelihood = −4632.2, random intercept variance for turtle ID = 0.00964, SD = 0.098; see Supplementary Table S2). In this model, the same interaction between SST and turtle size was significantly and negatively associated with inter-nesting interval (Type II Wald chi-square test: χ^2^(1) = 18.26, Bonferroni-adjusted *p* < 0.001; see Figure 3f), with larger turtles showing shorter intervals at higher SST. The detected positive correlation between CHL and inter-nesting intervals in this model (Type II Wald chi-square test: χ^2^(1) = 12.26, Bonferroni-adjusted *p* = 0.003; see Supplementary Table S2) may indicate that cooler periods (reflected by higher CHL) lengthen inter-nesting intervals.

### 3.3. Remigration Intervals

**Figure 4:**
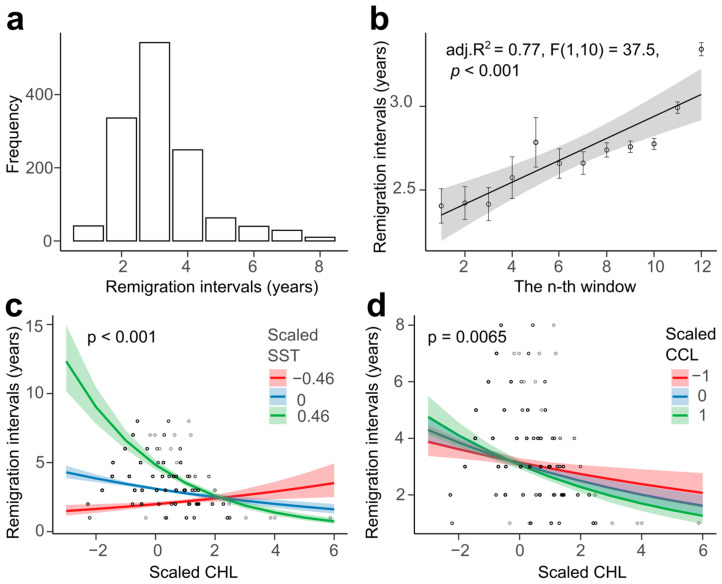
Patterns and predictors of remigration intervals in loggerhead turtle from Sal Island. (**a**) Distribution of remigration intervals. (**b**) Remigration intervals based on a rolling window approach of 5 nesting seasons rolling by 1 season step. (**c**) Two-way interaction of CHL and SST from foraging ground and (**d**) of CHL and turtle size between both predictors of remigration intervals. Shaded areas represent 95% confidence intervals.

Remigration intervals ranged from 1 to 8 years with an average of 3.18 ± 1.24 years (mean ± SD, *n* = 1310) (Figure 4a). We found a positive trend over time in remigration intervals (LM, adjusted R^2^ = 0.769, F(1,10) = 37.54, *p* < 0.001), suggesting turtles stay longer in their feeding ground now than they did at the start of the monitoring period (Figure 4b), even after accounting for interannual variation in nesting density (Supplementary Table S3).

Remigration interval was best explained by a model involving a three-way interaction between CHL at the foraging ground, residuals of SST at the foraging ground, and turtle size (GLM, AIC = 3887.6; residual deviance = 265.04 on 1199 degrees of freedom; see Supplementary Table S4). In this model, the interaction between SST and CHL was significant (ΔDeviance = 86.28, Bonferroni-adjusted *p* < 0.001; see Figure 4c). This result shows that higher ocean productivity at the foraging grounds shortens the foraging period. The interaction between CHL and turtle size was also significant (ΔDeviance = 7.41, Bonferroni-adjusted *p* = 0.045; see Figure 4d), showing that bigger turtles have shorter remigration intervals than smaller turtles as ocean productivity increases.

### 3.4. Clutch Frequency and Size

**Figure 5:**
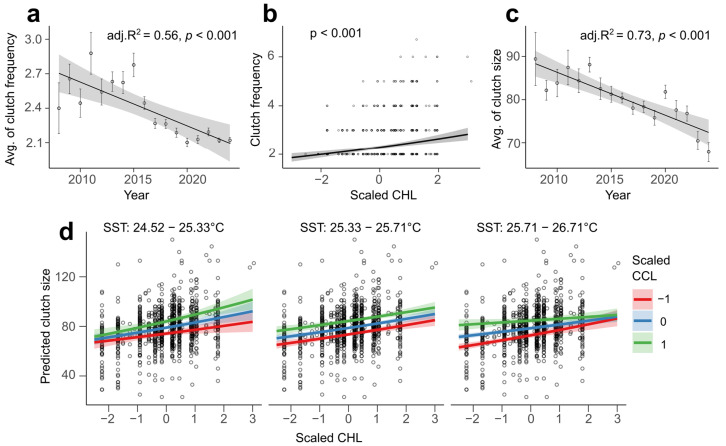
Trends and predictors of clutch frequency and size in loggerhead turtle from Sal Island. (**a**) Negative trend observed in clutch frequency between 2008 and 2024. (**b**) Clutch frequency is best predicted by chlorophyll-a (CHL) at foraging ground. (**c**) Declining trend in clutch size from 2008 to 2024. (**d**) Three-way interaction plot showing the trade-off between chlorophyll-a (CHL) at the foraging ground, turtle curved carapace length (CCL) and sea surface temperature (SST). GLMM: Bonferroni-adjusted *p*-value: 0.047. Shaded areas represent 95% confidence intervals.

Over the course of the monitoring period, the observed mean clutch frequency in this population was 2.31 ± 0.67 (mean ± SD, range = 2–8, *n* = 2126; see Supplementary Figure S6a), but we detected a decline over time (LM, adjusted R^2^ = 0.562, F(1,15) = 21.52, *p* < 0.001; see Figure 5a).

Clutch frequency was best explained by a model including an interaction between CHL at the foraging ground and turtle size (GLM, AIC = 6060; residual deviance = 306.43 on 2107 degrees of freedom; see Supplementary Table S5), with only CHL showing a significant effect (ΔDeviance = 29.38, Bonferroni-adjusted *p* < 0.001; see Figure S5b). When remigration interval was included in the model, the best model uncovered a positive correlation between clutch frequency and CHL in the foraging ground (ΔDeviance = 4.08, *p* = 0.043; see Supplementary Figure S6b and Table S6).

Over the course of the monitoring, the mean clutch size in this population was 79.03 ± 15.66 (mean ± SD, range: 23–150, *n* = 1003; see Supplementary Figure S6d). Similarly to the number of clutches, clutch size declined (LM, adjusted R^2^ = 0.732, F(1,15) = 44.71, *p* < 0.001; see Figure 5c). But this effect was not linked to an increased in neophytes over the years since they produced comparable clutch sizes to remigrants (Wilcoxon rank sum test, two-sided: W = 30,042, *p* = 0.10; see Supplementary Figure S6c). Clutch size declined by approximately one egg per week over the course of the season (LM, adjusted R^2^ = 0.04, F(1,5085) = 238.7, *p* < 0.001), with smaller turtles showing a faster rate of decline, suggesting a depletion of stored energy (Supplementary Figure S7).

Clutch size was best predicted by a model including a three-way interaction between SST at breeding ground, CHL at foraging ground and turtle size (GLM, AIC = 8185; residual deviance = 35.09 on 989 degrees of freedom; see Supplementary Table S7). In this model, the three-way interaction term was significant (ΔDeviance = 0.23, Bonferroni-adjusted *p* = 0.047; see Figure 5d). Specifically, independent of temperature, higher chlorophyll was associated with a higher clutch size. The effect was, however, levelled by turtle size, with larger turtles producing larger clutch sizes.

### 3.5. Trends in Body Size

Body size was significantly associated with reproductive parameters: larger females tended to have shorter inter-nesting (Figure 3) and remigration intervals (Figure 4) and larger clutches (Figure 5). Female size ranges from 60 to 111.8 cm, with the average of 79.6 ± 4.0 cm (*n* = 18,939). Mean female size declined over time (LM, adjusted R^2^ = 0.709, F(1,14) = 37.51, *p* < 0.001), suggesting an influx of neophytes. As expected, neophytes were smaller than remigrants (Wilcoxon rank sum: *p* < 0.0001) with both neophyte and remigrant shows decline in size (Supplementary Figure S8b). These shifts in size structure have direct implications for reproductive timing and output.

## 4. Discussion

Climate change has pervasive impacts on biological systems, influencing phenology and reproductive output across taxa [61,62,63]. These effects are especially pronounced in long-lived, capital breeding ectotherms such as sea turtles, whose reproductive success depends heavily on thermal conditions and resource availability. Using our extensive, continuous datasets for loggerhead turtles (*Caretta caretta*) nesting in Sal (Cabo Verde), we show clear relationships between environmental parameters and reproductive phenology and outputs: warmer SST was associated with earlier nesting phenology and shortened interval between clutches, while reduced productivity at foraging grounds correlated with increased remigration intervals and lowered clutch frequency and size. Together, these results demonstrate that climate warming acts through multiple, interacting pathways to influence reproductive timing and success in sea turtles, providing essential empirical evidence to guide climate-responsive conservation of one of the world’s largest loggerhead populations.

### 4.1. Phenology and Season Length

Although no long-term change in nesting phenology was detected, we found that the timing of reproduction was strongly linked to ocean temperature. Warmer SST at the foraging ground in the months preceding the nesting season was associated with an earlier seasonal start, suggesting that females begin migrating sooner and/or shorten their pre-nesting residence at the breeding site [64]. Once the nesting season started, higher SST at breeding beaches was associated with earlier median and end of the season, likely accelerating the rate of physiological processes involved in ovulation and egg development [31]. These findings support the idea that both foraging and breeding SST influence phenological timing [18,19,20,21,65], even though the direction and magnitude of such responses can vary among populations [66].

Interestingly, while temperature shaped the timing of phenology, it did not affect the total season length, unlike in other loggerhead populations at Canaveral National Seashore on the Atlantic coast of central Florida [65] and at northern Gulf of Mexico [18]. Instead, season length was best predicted by the timing of start: years with later starts also tended to end earlier. Because both variables derive from percentile-based definitions, part of this relationship may reflect statistical coupling, yet biological explanations remain plausible. Warmer years were characterized by faster increases in nesting activity early in the season, followed by slower declines later in the year. Such asymmetric seasonal dynamics suggest that higher temperatures not only enhance reproductive readiness at the start of the season but also prolong favourable nesting conditions into the late season. Access to nearshore warmer water (i.e., facilitated by reduced human presence) has been linked to an increase in the number of clutches laid per season, indicating an extension of nesting period under favourable environmental conditions [67]. Furthermore, warming can lengthen the thermal window for successful incubation and hatchling emergence [15,41]. Collectively, these results reveal that temperature does not simply shift nesting seasons earlier or later, but can reshape their structure, compress the start and extend the tail end of reproductive effort.

### 4.2. Timing of Arrival and Estimates of Population Size

Continuous tagging over nearly two decades also provided insights into nesting season dynamics. Generally, females reached nesting beaches for their first nest throughout a major part of the nesting season [17,66]. Indeed, we observed that the overall proportion of newly recorded individuals declined steadily after mid-season but never ceased entirely, indicating sustained recruitment, with a detected increase in newly recorded individuals suggesting continuous arrival to the nesting grounds. This could imply that since turtles use different feeding grounds, timing to initiate the migration is asynchronous and locality-specific [68], making them arrive at different times. It is important to note that part of this pattern may be reflected by the detection of previously untagged/undetected turtles rather than being solely due to late-season first-time arrivals. Recapture rates remained stable over the monitoring period except during the 2020–2021 COVID-impacted years. On average, patrols tagged 22% of daily nesting turtles, translating to roughly a 53% probability of detecting an individual at least once over three clutches within a season. Using this probability window, we can start to estimate the number of turtles, with Sal Island alone hosting approximately 9000 to 17,000 nesting females at the peak of the nesting trend. As Sal is the second-largest aggregation in Cabo Verde, these figures reaffirm that Cabo Verde supports one of the largest loggerhead nesting populations globally [49] and underscore the conservation importance of maintaining the integrity of this rookery [69].

### 4.3. Inter-Nesting Intervals

Inter-nesting intervals, the time between successive clutches, integrate both physiological and environmental constraints on reproduction [29,38]. In this population, intervals ranged widely (9–84 days) with a multimodal distribution. The longer intervals recorded early in the season corresponded to cooler temperatures and earlier arrival timing, supporting the hypothesis that egg maturation is thermally limited [27,70]. Restricting analyses to turtles where we are unlikely to have missed a nesting event (9–20 days) revealed a clear negative correlation between SST and interval length. The negative effect of SST on inter-nesting intervals has been widely documented across several sea turtle species [27,29,30,71]. In our study, we also found that this relationship was size-dependent. Larger females exhibited shorter inter-nesting intervals in warmer waters but longer intervals during cooler periods, suggesting the influence of thermal inertia in this ectothermic species [39]. Under warmer conditions, their elevated and sustained body temperatures may accelerate egg development and reduce the resting period between clutches. Smaller turtles, on the other hand, may gain and lose heat more under fluctuating conditions. These interacting effects suggest that thermal conditions and morphology jointly shape reproductive pacing. If the trend toward smaller average female size observed since 2014 continues [72], the population may experience longer mean inter-nesting intervals, potentially lowering annual fecundity.

### 4.4. Remigration Intervals

Although some individuals exhibited unusually short or long remigration intervals, most turtles typically returned to nest after about three years of foraging. It is consistent with other Atlantic populations [73] but longer than those in the Pacific [74]. We detected an increasing trend over time, indicated that females now spend longer at foraging ground before returning to breed. Although such a trend may partially reflect sampling effort relative to nesting density, we found no evidence that variations in nest abundance confound this relationship. The increased remigration interval is consistent with the continuous decline in ocean productivity detected over the course of this study. As expected, warmer years that reduced ocean productivity led to longer remigration intervals. Comparable patterns have been reported in green turtles from Costa Rica, where low ocean productivity during El Niño events delayed remigration [75].

Furthermore, the influence of resource availability on remigration intervals varied with turtle size, with smaller individuals being more affected by limited resources than larger ones [74]. This mirrors Japanese populations, where small, oceanic foragers have longer remigration intervals than large, benthic-foraging females [46,74]. The progressive lengthening of remigration intervals in Cabo Verde therefore likely reflects declining productivity in combination with demographic shifts toward smaller females. Monitoring of resource availability, and possibly feeding ground shift is important, as these extended non-breeding intervals could slow population growth and alter generational turnover.

### 4.5. Clutch Frequency and Size

We discovered that the observed mean clutch frequency of 2.3 clutches per female lies at the lower end of global estimates derived from recapture and satellite telemetry studies, with 3.5–4.5 clutches in average in southeast USA [76,77]; up to 5.4 in Oman [78]; 2.4–3.8 in Greece [79]; or 1.8 in Cyprus [56]. In previous estimates from Boa Vista in Cabo Verde, the observed mean clutch frequency was lower (1.4), ranging from one to six [28]. Such difference may stem from Cabo Verde sea turtles being smaller than turtles from other Atlantic populations, and being more comparable in size to Mediterranean populations [53]. Furthermore, the clear positive association between clutch frequency and foraging ground productivity suggests a biological mechanism. Females foraging in productive waters can accumulate larger fat reserves [42], sustain near-complete fasting during the nesting season and produce a greater number of clutches. The absence of a difference between neophytes and remigrants implies that energy availability, rather than age or reproductive experience, governs clutch frequency.

As expected, clutch size was strongly correlated with female size, e.g., refs. [43,56,80]. Clutch size also correlated with foraging ground ocean productivity [41]. Our results are consistent with previous isotopic studies that showed positive correlations between δ^15^N and δ^13^C signatures (indicators of trophic level and foraging habitat productivity) and clutch size in loggerheads [42]. Here, we add the knowledge that during the warmest years, the positive correlation between size and clutch size weakened, suggesting that thermal stress or energetic limitations constrains investment in reproduction.

The overall decline in clutch size through time likely results from both environmental and demographic processes, such as reduced foraging productivity, and a growing proportion of smaller, first-time nesters since 2013 [72]. Because clutch size contributes directly to population fecundity, such reductions may have compounding effects on future recruitment, even in this currently growing population. Monitoring shifts in female body size structure therefore provides an important early warning indicator of population-level reproductive change. Ultimately, monitoring in the feeding ground should continue and investigating migration routes will provide better guidance for future conservation efforts.

## 5. Conclusions

All five hypotheses were supported. Using 17 years of continuous monitoring and tagging data, we demonstrate that the phenology of loggerhead nesting in Sal, Cabo Verde, is associated with sea surface temperature (SST) in both feeding and breeding grounds. Specifically, warmer years advanced the start, median, and end of the nesting season. Inter-nesting intervals were shorter during warmer SST, particularly in larger females. There has been a significant decline in ocean productivity in the feeding grounds, which is associated with longer foraging periods and reduced clutch sizes. Furthermore, with the decline in body size in the population due to neophyte influx, reproductive output decreased further, as smaller females produced smaller clutches. 

From a conservation perspective, our findings highlight the need to incorporate climate-driven variability in phenology and reproductive output into management and monitoring strategies for loggerhead turtles in Cabo Verde. Continued long-term monitoring at nesting beaches remains essential but should be complemented by sustained observation of foraging grounds, where declining productivity appears to have cascading effects on remigration intervals, clutch frequency, and clutch size. Future research involving systematic assessment of foraging grounds and migration routes will be essential to identify critical feeding areas, detect shifts in habitat use, and prioritize protection of key productivity hotspots. Given the observed demographic shift toward smaller females and its implications for fecundity, monitoring changes in female body-size distribution remains critical, as these metrics provide early-warning indicators of population-level reproductive change under ongoing ocean warming and declining productivity.

## Data Availability

The original contributions presented in this study are included in the article/Supplementary Materials. Further inquiries can be directed to the corresponding author.

## Supplementary Materials

The following supporting information can be downloaded at https://www.mdpi.com/article/10.3390/ani16040552/s1, Figure S1: Hypothesized chronogram of reproductive activities of loggerhead nesting on Sal Island, Cabo Verde; Figure S2: Monthly observed nest counts in all seasons shows when the typical nesting season starts; Figure S3: Season length and sea surface temperature (SST) correlations; Figure S4: Inter-nesting intervals and recapture rates; Figure S5: Inter-nesting intervals trend and structure among different status of individuals; Figure S6: Clutch frequency and size trend; Figure S7: Clutch size trend throughout the season in loggerhead population nesting in Sal Island; Figure S8: Size trend in loggerhead population nesting in Sal Island; Table S1: Summary of the model predicting inter-nesting intervals with all data set (9–84 days); Table S2: Summary of the model predicting inter-nesting intervals between 9–20 days; Table S3: Summary of the model predicting remigration intervals trends over time accounting for annual variation in nest counts; Table S4: Summary of the model predicting remigration intervals; Table S5: Summary of the model predicting clutch frequency excluding remigration intervals as the predictor; Table S6: Summary of the model predicting clutch frequency including remigration intervals as one of the predictors; Table S7: Summary of the model predicting clutch size. SSTbg = SST at breeding ground, CHLfg = CHL at foraging ground.
