# Supplementary material for: Warming and Change in Ocean Productivity Alter Phenology of an Expanding Loggerhead Population in Cabo Verde
Source: Animals (Basel). 2026 Feb 11;16(4):552. doi: 10.3390/ani16040552 (PMC12937436; doi:10.3390/ani16040552)
Supplement: Supplementary file 1 [file animals-16-00552-s001.zip › Supplementary files/Supplementary Figures.pdf]

Supplementary Figures

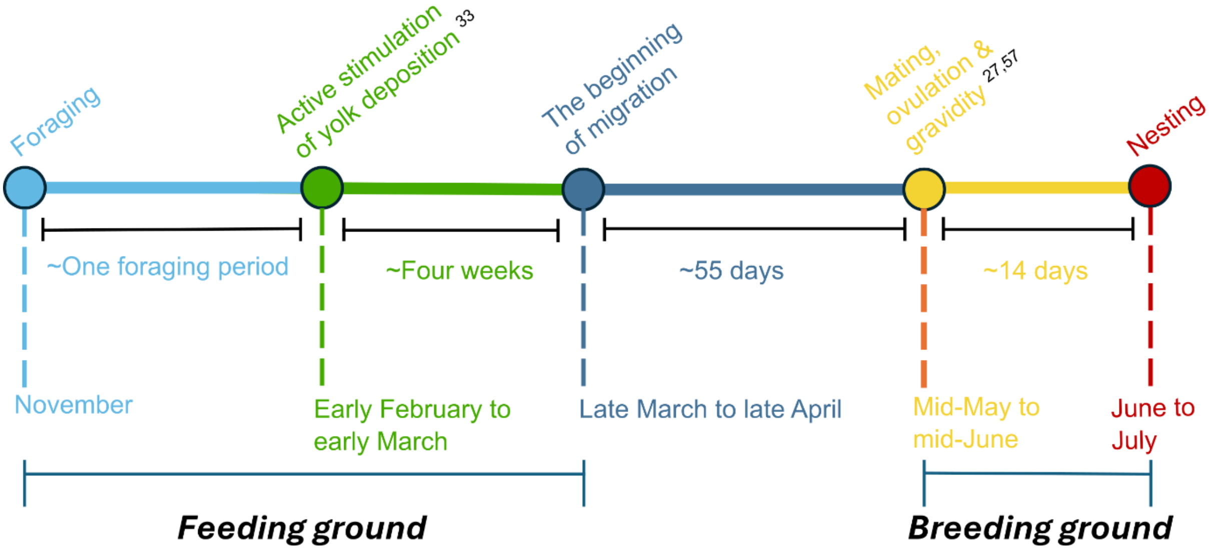

Supplementary Figure S1. Hypothesized chronogram of reproductive activities of loggerhead nesting on Sal Island, Cabo Verde. Superscript numbers are references.

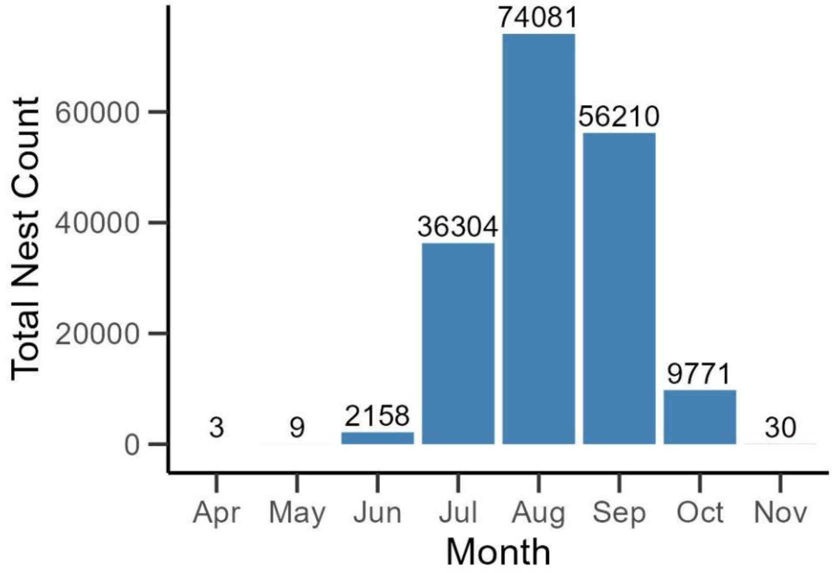

Supplementary Figure S2. Monthly observed nest counts in all seasons shows when the typical nesting season starts. Numbers above bars represents the observed nest counts, excluding nest counts from extrapolated data/irregularly patrolled beaches.

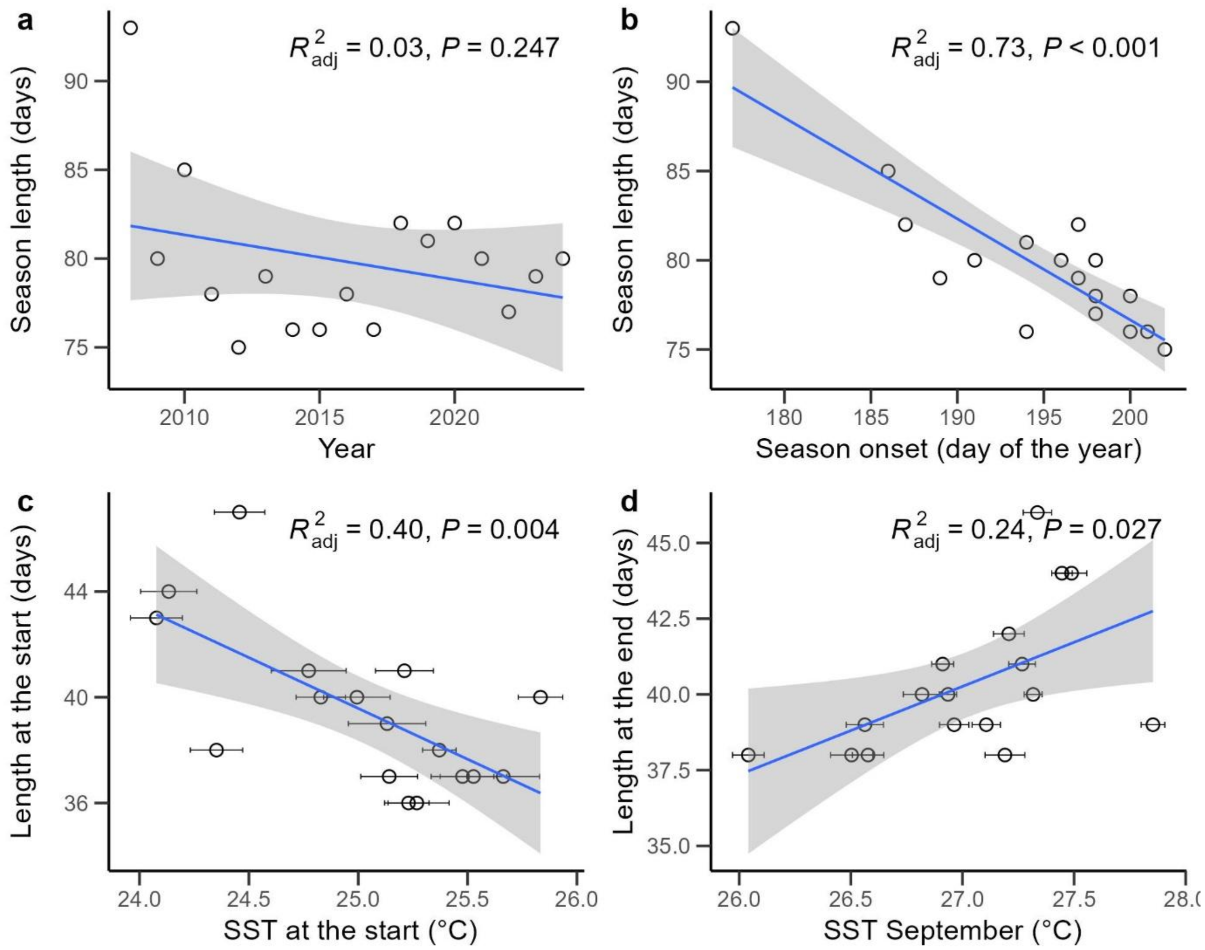

Supplementary Figure S3. Season length and sea surface temperature (SST) correlations. (a) Season length has not shifted over the years, and (b) is predicted by the start of the season (linear model) especially later start would result in shorter nesting season. (c) The early phase (from 5% of nest count to 50%) was best predicted by SST during the early phase and (d) the end phase (from 50% of nest count to 95%) was best predicted by SST during September.

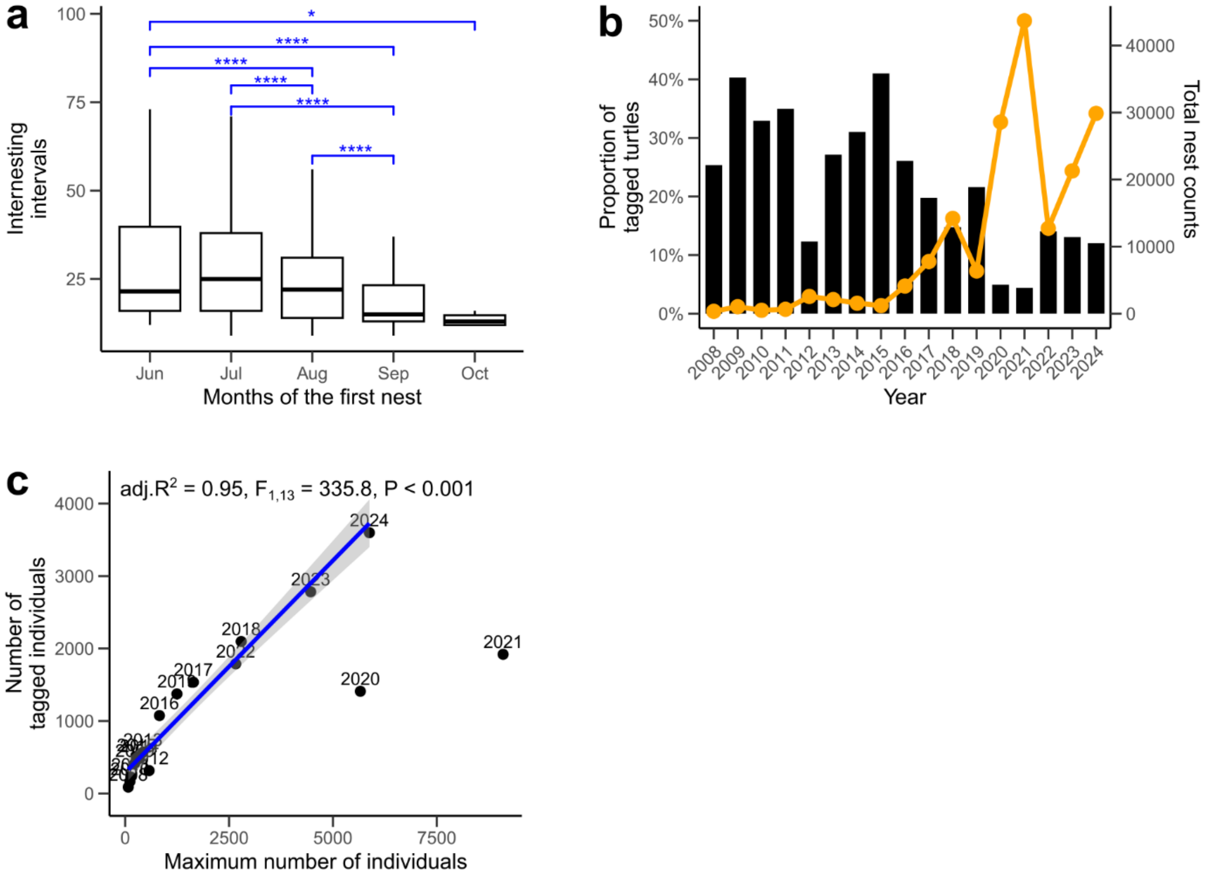

Supplementary Figure S4. Inter-nesting intervals and recapture rates. (a) Inter-nesting intervals in the early season were longer than later in the season. Blue bars and stars denote significant differences between months, * $p < 0.05$, **** $p < 0.0001$. (b) Probability of detecting/deploying a tag over the years. The primary y-axis is the number of tagged turtles over the total nest counts. Secondary y axis is the total nest counts. Black bars show the proportion of tagged turtles. Orange line shows total nest counts. and (c) recapture rates increase as the number of females nesting increase. Maximum number of individuals is estimated by using 14-day rolling window approach.

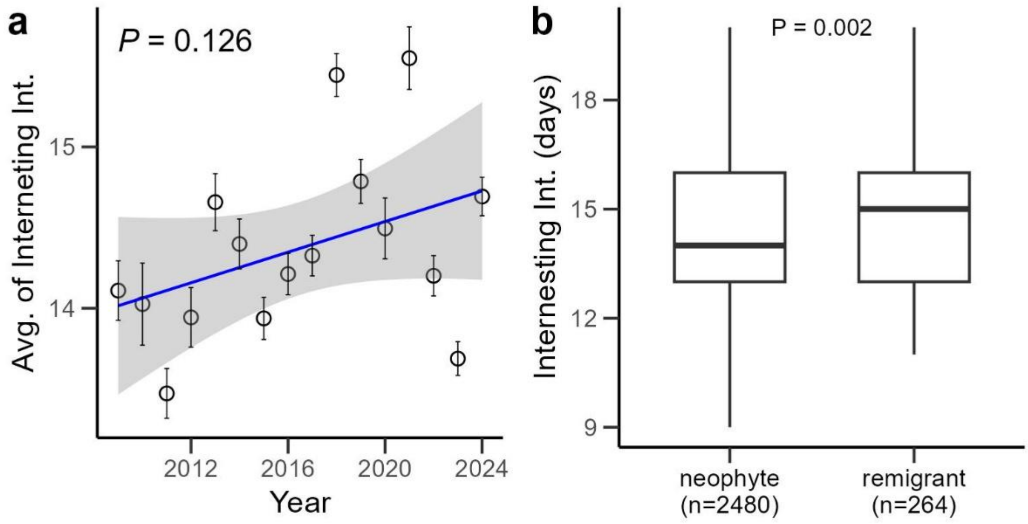

Supplementary Figure S5. Inter-nesting intervals trend and structure among different status of individuals. (a) Inter-nesting intervals have not shifted over the years and (b) there was a significant difference in inter-nesting intervals between neophytes and remigrants.

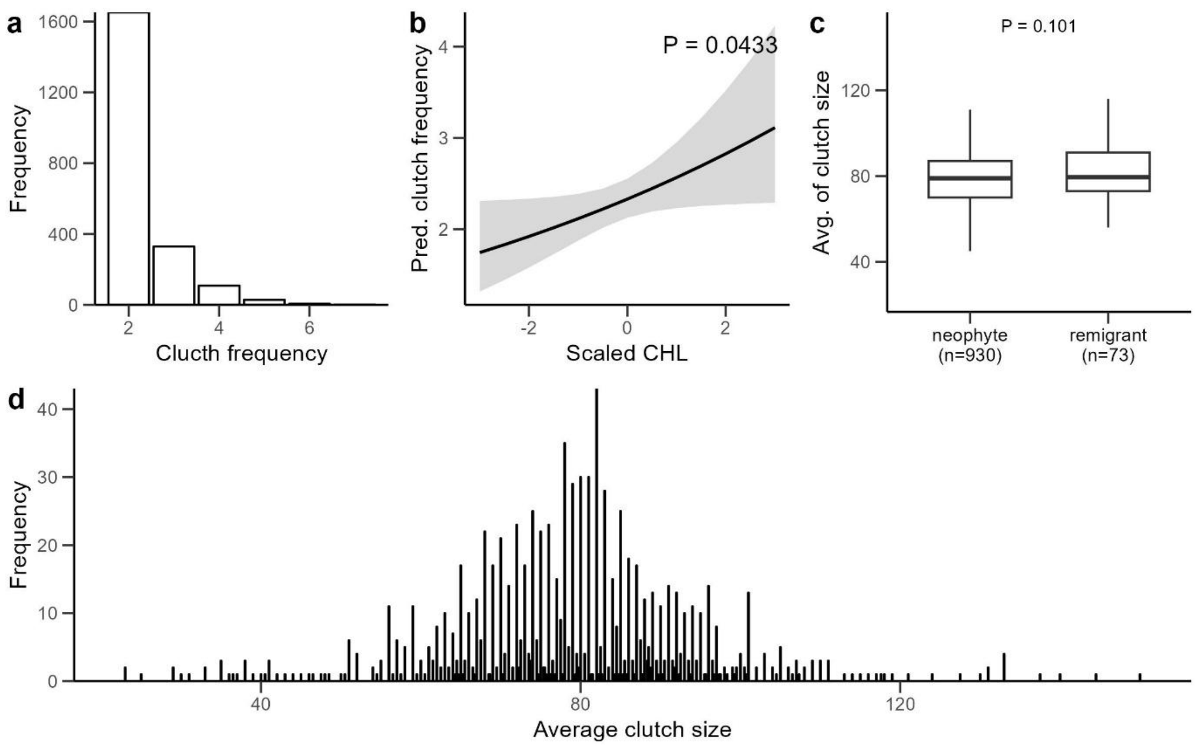

Supplementary Figure S6. Clutch frequency and size trend. (a and d) showing distribution of clutch frequency and size. (b) Clutch frequency was best predicted by chlorophyll-a concentration at the foraging ground when including remigration intervals as a predictor. (c) there was no difference between neophytes and remigrants in clutch size.

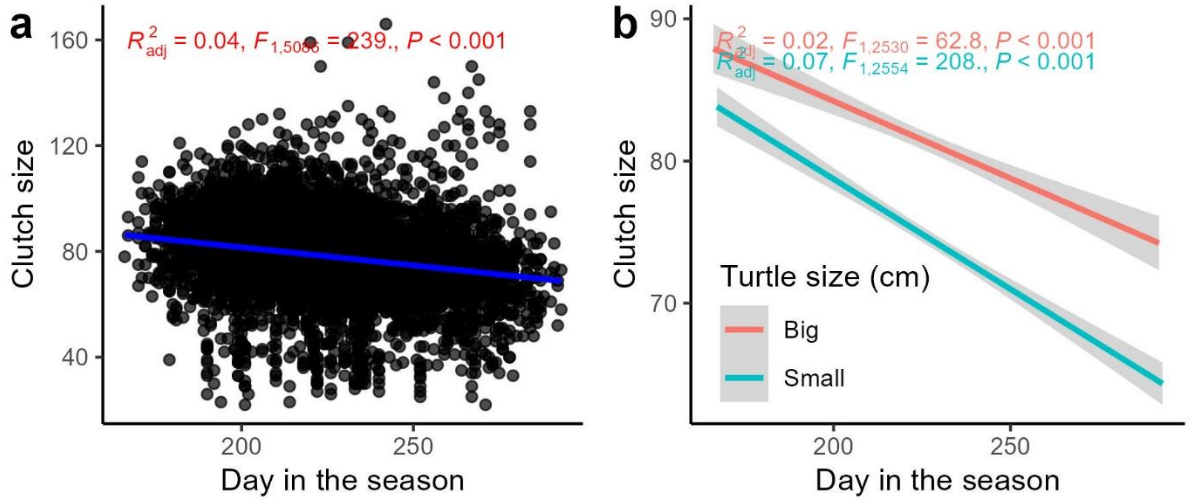

Supplementary Figure S7. Clutch size trend throughout the season in loggerhead population nesting in Sal Island. (a) Overall clutch size trend including both small and big size turtle. (b) The declining rate in smaller turtles is faster than the bigger turtles. Small = 44.8 - 79.1 cm, Big = 79.17 - 104.77 cm.

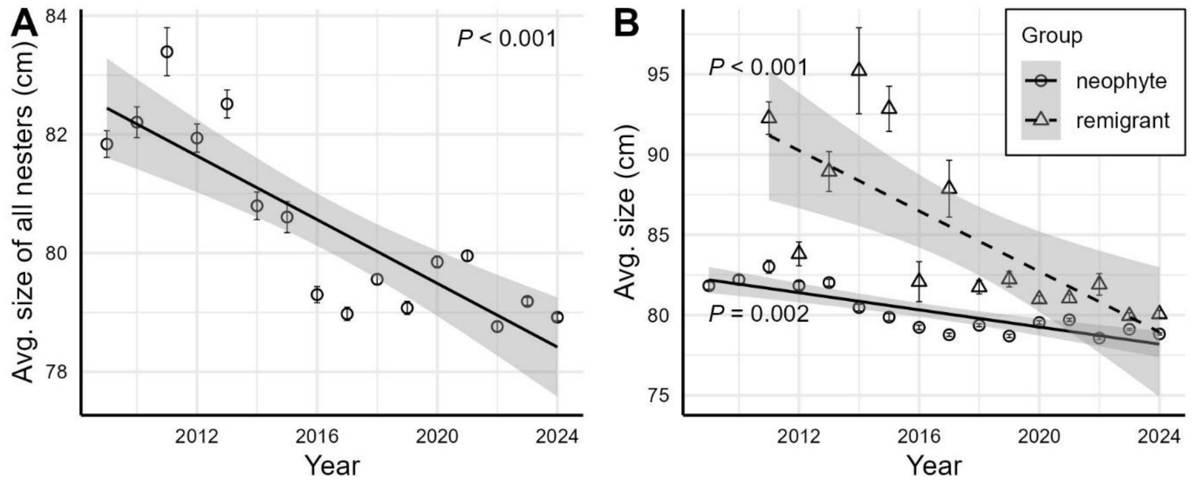

Supplementary Figure S8. Size trend in loggerhead population nesting in Sal Island. (A) Overall trend including both remigrant and neophyte. (B) Size trend in neophyte and remigrant.
